# Gamble game: a pilot mixed-methods study on board games for adolescent gambling prevention in the school setting

**DOI:** 10.3389/fpsyg.2026.1756454

**Published:** 2026-04-02

**Authors:** Paola Cardinali, Stefania Guasco, Fabio Boero, Martina Olcese

**Affiliations:** 1 Department of Human and Social Science, Universitas Mercatorum, Rome, Italy; 2 Digital Clinical Psychology Unit ASL1 SSR, Imperia, Italy; 3 L’Ancora Cooperative- Imperia, Imperia, Italy; 4 Department of Educational Science, University of Genoa, Genoa, Italy

**Keywords:** adolescent, board games, cognitive distortions, experiential learning, gambling prevention, participatory education

## Abstract

Adolescent gambling is an increasing public health concern, posing risks to emotional and social development. Despite legal prohibitions, the normalization of gambling through advertising and informal contexts promotes cognitive distortions and unrealistic expectancies among youth. Evidence-based preventive programs using participatory and engaging methods are therefore crucial. This pilot study evaluated Game in Lab, a cluster-randomized school-based intervention co-developed by universities, local health services, and the third sector. The program employed board game activities and guided debriefing to enhance awareness, critical thinking, and emotional reflection on risk, aiming to deconstruct gambling-related misconceptions. A total of 210 students participated, and 129 completed pre- (T0) and post-intervention (T1) measures. Classes were randomized to intervention or control conditions. Quantitative outcomes included the Gambling Related Cognitions Scale (GRCS) and Gambling Expectancy Questionnaire–Modified (GEQ-MOD); qualitative data were collected through three focus groups with intervention students. Descriptive analyses indicated reductions in cognitions and expectancies across groups, but cluster-adjusted ANCOVAs did not confirm additional effects for the intervention group, which maintained slightly higher post-test scores. Conversely, thematic analysis showed increased critical thinking about probability, greater emotional awareness, and improved understanding of gambling risks. Overall, findings suggest that board games are a feasible and engaging tool for school-based gambling prevention. While quantitative results were mixed, qualitative evidence highlights their potential to foster reflection and awareness among adolescents. Future larger-scale studies with follow-ups are recommended.

## Introduction

Adolescence is a developmental stage characterized by heightened vulnerability to risk-taking behaviors, including gambling, which can have negative psychological, academic, and relational consequences (Zhang and Wang, 2025). In Italy, epidemiological studies indicate a marked North–South gradient, with higher prevalence rates of at-risk and problem gambling among adolescents living in regions with greater income inequality (Canale et al., 2015). Perceived support from parents and teachers has been identified as a protective factor in this context (Davis et al., 2025). From a psychological perspective, gambling behaviors in adolescence are sustained by cognitive distortions, such as misperceptions of randomness, the illusion of control, and predictive beliefs, as well as by gambling-related expectancies. Positive expectancies (e.g., pleasure, excitement, monetary gain) have been linked to increased gambling frequency and problem severity, while negative expectancies (e.g., over-involvement, emotional impact) predict more severe levels of gambling problems (Donati et al., 2022). Despite the legal prohibition for minors, gambling has become increasingly normalized through advertising, online platforms, and informal social contexts, which reinforce these distortions and unrealistic expectations (Bolat et al., 2025).

Schools represent a crucial setting for prevention because they allow systematic access to large populations of adolescents and can integrate educational strategies with the protective role of teachers and families. Empirical evidence shows that brief school-based programs aimed at fostering probabilistic reasoning and correcting biases can significantly reduce gambling-related cognitive distortions (Miller et al., 2025). These interventions, often structured in short modules delivered during regular school hours, focus on concepts such as independence of random events and the fallacy of predictive control, with the goal of transferring knowledge from “cold” probabilistic tasks to “hot” gambling-related contexts (Primi and Donati, 2022). This body of research highlights the potential of educational interventions delivered in the school setting to promote critical thinking and resilience against gambling behaviors in youth.

Beyond traditional lectures, interactive approaches have been increasingly recognized as effective strategies to promote health-related knowledge and behavior change in adolescents. Active methodologies that enable students to be involved, responsible, and committed to their teaching and learning processes acquire particular relevance in terms of facilitating competency development in the classroom (Jones, 2017). Board games represent a concrete example of such methodologies, as they promote experiential and participatory learning, foster collaboration among peers, and require players to take responsibility for decisions and their consequences. Recent evidence supports the educational potential of board games in different domains, from reducing stigma and promoting mental health awareness (Respati et al., 2024), to enhancing executive functions and academic skills (Vita-Barrull et al., 2024), and strengthening emotional competences (Dell'Angela et al., 2020). A systematic review has documented that board games in health education produce substantial effects on knowledge acquisition and moderate effects on behaviors and health outcomes (Gauthier et al., 2019). Board games create an engaging and participatory learning environment, stimulating peer interaction and reflection. In the gambling domain, they provide a promising avenue for addressing cognitive distortions and gambling expectancies in an experiential way, linking playful activities with guided discussions to enhance awareness of randomness, risk, and self-regulation (López Jiménez et al., 2025). By combining ludic dynamics with educational objectives, board games can support adolescents in developing a more critical and conscious approach to gambling.

Despite these promising findings, few studies have evaluated the use of board games as structured preventive interventions specifically targeting gambling in adolescence (Angelini et al., 2024). Existing school-based programs have mainly focused on traditional educational activities, with limited integration of experiential and interactive components (Talebi and Bazrafshan, 2025). Moreover, most available evidence relies on quantitative outcomes, without systematically combining students’ subjective perspectives to capture how preventive experiences are understood and internalized. To address these limitations, the Game in Lab project was developed as a cluster-randomized school-based intervention using board games and guided debriefing sessions to stimulate probabilistic reasoning, critical reflection, and awareness of gambling-related cognitions and expectancies. The project adopts a mixed-methods approach, integrating quantitative measures with qualitative focus groups, in order to assess both the effectiveness of the training on cognitive outcomes and the processes of change as perceived by students. This design aims to contribute novel evidence on the potential of board games as preventive tools in adolescence, advancing knowledge on how playful and participatory formats can support gambling prevention in school contexts.

## Methods

### Participants

A total of 210 students from Italian secondary schools were initially enrolled in the study. Of these, 183 completed the baseline assessment (T0) and 160 completed the post-intervention assessment (T1). A total of 129 students (61.4%) provided complete data at both baseline (T0) and post-intervention (T1) and were included in the main analyses (Table 1). Attrition was primarily due to student absence on the day of data collection, or student incomplete participation in the training sessions.

**Table 1:** Participant flow across data collection waves.

Stage	*N*
Students enrolled	210
Completed baseline assessment (T0)	183
Completed post-intervention assessment (T1)	160
Provided complete data at both waves	129

To examine potential attrition bias, baseline gambling expectancies and gambling-related cognitions were compared between students who completed both waves and those with missing post-intervention data. Independent-samples t tests showed no significant differences between completers and non-completers for gambling expectancies, *t*(181) = −0.67, *p* = 0.503, or gambling-related cognitions, *t*(181) = −1.04, *p* = 0.301.

Students were nested within 12 classes, which represented the unit of randomization. Six classes were assigned to the intervention condition and six to the control condition.

Entire classes were invited to participate, and students who returned parental consent forms and provided individual assent were included in the study. Participation was voluntary and anonymous. The study protocol was approved by the Mercatorum Ethical Committee (Prot. 0615/February 2024), and all procedures complied with the Declaration of Helsinki. Schools represented different educational tracks, including both humanities- and science-oriented high schools as well as technical institutes.

### Design

The research was designed as a cluster-randomized controlled trial (RCT). Randomization occurred at the class level: entire classes were randomly assigned to either the intervention group (*Game in Lab* training) or the control group (no training). Data were collected at two time points: pre-intervention (T0) and post-intervention (T1). A mixed-methods approach was adopted: alongside quantitative measures, three focus groups were conducted to explore students’ subjective experiences and perceived changes.

### Procedure

The *Game in Lab* training was implemented over three weekly classroom sessions, each lasting approximately 90 min. The sessions were delivered by trained facilitators and followed in a structured format consisting of two core components: board game play and guided debriefing discussions.

During board game activities, students played selected games designed to stimulate reflection on chance, randomness, decision-making under uncertainty, and self-regulation. These games provided experiential opportunities for students to confront situations where probabilistic reasoning and risk evaluation were required (Table 2).

**Table 2:** Games used in the training.

Board game	Short description of game idea	Core dynamics	Educational objectives	Knowledge elements to be learned
*Perudo* (Asmodee)	Dice game of hidden rolls and bluffing where players bet on the total number of dice showing a given value.	Dice rolling, bluffing, uncertainty management, elimination of players.	Stimulate probabilistic reasoning and strategic thinking; highlight how beliefs influence decisions.	Randomness of outcomes; independence of events; gambler’s fallacy.
*Las Vegas* (Ravensburger)	Players place dice in casinos to win money, competing for majority control.	Dice placement, competition for majority, risk vs. reward.	Encourage resource management and reflection on impulsivity.	Majority dynamics; influence of context and rules; evaluation of probabilities vs. risks.
*Deep Sea Adventure* (Oink Games)	Players dive together with shared oxygen, trying to collect treasures and return safely.	Push-your-luck, shared resources, collective consequences.	Foster awareness of shared risk and cooperative decision-making.	Trade-off between risk and reward; collective vs. individual interest; escalation of risk-taking.
*Cannot Stop* (Print & Play)	Dice game where players try to advance on number columns, risking to continue or stop.	Push-your-luck, probability combinations, escalation of risk.	Teach probability calculation; promote reflection on rational vs. impulsive choices.	Independence of rolls; gambler’s fallacy; escalation of commitment.
*Voyages* (Postmark Games)	Roll-and-write game where players use dice to explore maps and optimize resources.	Dice-driven exploration, resource allocation, long-term strategy.	Differentiate between skill and chance; promote critical thinking.	Distinction between luck and strategy; limits of control over random events; probability in planning.

Each session was followed by guided debriefing, during which facilitators encouraged students to analyze their gameplay experience critically. The discussions explicitly linked game dynamics to gambling-related cognitions and expectancies, addressing common cognitive biases (e.g., illusion of control, gambler’s fallacy) and reinforcing the understanding of randomness and probability. Debriefings were interactive and dialogical, fostering peer exchange and promoting awareness of gambling risks in everyday life.

The training sessions were conducted between March and May 2024.

Quantitative data were collected at baseline (T0) and immediately after the training (T1) using validated self-report measures of gambling-related cognitions, expectancies, and behaviors. To complement these outcomes and explore students’ subjective perspectives, three focus groups were conducted after the intervention with a purposive sample of participants (*n* = 25). The qualitative component provided insights into students perceived learning, emotional experiences, and processes of change. In line with a mixed-methods triangulation design (Aresi et al., 2025), qualitative findings were integrated with quantitative results to build a comprehensive understanding of the effects and mechanisms of the intervention.

### Quantitative measures

The self-report questionnaire included the following measures:

- Sociodemographic information

 Students reported their age, gender, school type (humanities- or science-oriented high school, technical institute, vocational school), and ethnicity (Italian, first-generation migrant, second-generation migrant).

- Gambling outcome expectations

 The *Gambling Expectancy Questionnaire – Modified* (GEQ–MOD; Donati et al., 2022) was used to assess gambling-related expectancies. The scale is composed of 19 items rated on a 5-point Likert scale from 1 (*totally disagree*) to 5 (*totally agree*). Seven items measure Enjoyment/Arousal (e.g., “Gambling would make you feel more relaxed”), three items assess Self-Enhancement (e.g., “Gambling would make you feel more accepted by people”), and three items measure Money (e.g., “Gambling would make you get rich”). Among the remaining items, three measure Over-Involvement (e.g., “Gambling would make you want to gamble more and more”) and three Emotional Impact (e.g., “Gambling would make you feel guilty”). In the present study, the scale demonstrated good reliability (Cronbach’s *α* = 0.88).

- Erroneous gambling cognitions

 The *Gambling Related Cognitions Scale* (GRCS; Raylu and Oei, 2004) was used to assess adolescents’ distorted cognitions about gambling. The scale is composed of 23 items rated on a 7-point Likert scale from 1 (*strongly disagree*) to 7 (*strongly agree*). It comprises five subscales: four items measure Expectancies (e.g., “Gambling makes things seem better”), four items measure Illusion of control (e.g., “I have specific rituals and behaviours that increase my chances of winning”), six items assess Predictive control (e.g., “Losses when gambling are bound to be followed by a series of wins”), four items measure Interpretative bias (e.g., “Relating my winnings to my skill and ability makes me continue gambling”), and five items assess Perceived inability to stop gambling (e.g., “My desire to gamble is so overpowering”). In the present study, the total scale showed excellent internal consistency (Cronbach’s *α* = 0.92).

### Qualitative measures

To facilitate a typical focus group setting, intervention classes were asked to select a maximum of 8–10 students to participate in the discussion. A total of three focus groups (*n* = 25) were conducted at the schools between November and December 2024. Each focus group was facilitated by a psychologist with experience in qualitative research and in the delivery of school-based prevention programs.

Discussions followed by a semi-structured script composed of broad guiding questions and open-ended prompts. The topic guide explored students’ perceptions of the training and its impact on their cognitions and attitudes toward gambling.

Each focus group lasted approximately 75 min and was audio-recorded and transcribed verbatim. Across the three discussions, participants’ accounts showed substantial convergence, with similar themes recurring across groups. This repetition of patterns suggested that thematic saturation had been reached.

### Data analysis

Quantitative data were analyzed using Jamovi 3.0. (Version 3.0; jamovi, 2024). Primary analyses were conducted through ANCOVA with cluster adjustment, in which post-test scores were regressed on group (training vs. control), controlling for baseline scores, and including a random intercept for class to account for the cluster-randomized design. This model was selected to account for the cluster-randomized design and to maximize statistical power. Because randomization occurred at the class level, models included a random intercept for class to account for the non-independence of observations within clusters. Results are reported as unstandardized estimates with standard errors, *t* values, *p* values, and 95% confidence intervals. Descriptive statistics were computed for sociodemographic variables and questionnaire scores. Internal consistencies (Cronbach’s *α*) were calculated for each measure. Preliminary 2 × 2 mixed ANOVAs (Time × Group) were also conducted as exploratory analyses to examine overall time effects before accounting for clustering.

Qualitative data from the focus groups were analyzed using reflexive thematic analysis (Braun and Clarke, 2006). Transcripts were read multiple times to ensure familiarity, then systematically coded to identify recurrent patterns in participants’ accounts. The first author conducted the initial coding of the transcripts. Coding decisions and theme development were subsequently discussed within the research team to support reflexivity and ensure consistency in interpretation. When differences in interpretation emerged, these were discussed until a shared understanding was reached. Codes were organized into broader themes reflecting students’ critical thinking about chance and probability, awareness of gambling-related risks, and recognition and regulation of emotional reactions during gameplay. Illustrative quotes were selected to exemplify each theme. Findings were integrated with quantitative results through a triangulation perspective, to provide a more comprehensive understanding of the effects and processes activated by the intervention.

### Results

A total of 129 students provided complete data at both baseline and post-test (Table 3). The sample included 70 males (54.3%) and 59 females (45.7%). Most participants were Italian (74.6%), while 11.9% were first-generation migrants and 13.5% were second-generation migrants. The mean age was 16.0 years (SD = 1.1, range 14–19). Students were enrolled in different school tracks: 31.8% attended humanities- or science-oriented high schools, 38.8% technical institutes, and 29.5% vocational schools.

**Table 3:** Descriptives of the sample.

Variable	*n*	%	*M* (SD)	Range
Gender				
Male	70	54.3%		
Female	59	45.7%		
Ethnicity				
Italian	94	74.6%		
First-generation migrant	15	11.9%		
Second-generation migrant	17	13.5%		
School type				
High school	41	31.8%		
Technical institute	50	38.8%		
Vocational school	37	29.5%		
Age (years)	129	100%	16.0 (1.1)	14–19

Table 4 reports mean and standard deviations of the outcome measures by group and time.

**Table 4:** Descriptive statistics for gambling-related measures by group and time.

Measure	Group	Pre-test M (SD)	Post-test M (SE, EMM)
Gambling Expectancies (GEQ-MOD total)	Control (*n* = 43)	2.34 (0.59)	2.20 (0.11)
	Training (*n* = 86)	2.56 (0.65)	2.45 (0.08)
Gambling Cognitions (GRCS total)	Control (*n* = 43)	2.11 (1.06)	1.85 (0.13)
	Training (*n* = 86)	2.42 (1.16)	2.25 (0.09)

Pre-test values are observed means (SD). Post-test values are estimated marginal means (SE) adjusted for baseline scores with random intercepts for class.

Both groups showed lower scores at post-test compared to baseline, suggesting an overall reduction in gambling-related expectancies and cognitions over time.

Preliminary analyses conducted with a 2 × 2 mixed ANOVA (Time × Group) suggested a reduction in gambling-related cognitions and expectancies in the intervention group compared to controls. However, these results did not take into account the clustered design of the study. To provide a more rigorous test of the intervention effects, we subsequently performed secondary analyses using cluster-adjusted ANCOVA models with class as random intercept and baseline scores as covariates. Cluster-adjusted ANCOVA models were conducted to test whether the intervention group showed greater reductions compared to the control group, controlling baseline scores (Table 5). Results indicated no significant effect of training on gambling expectancies [*b* = 0.25, SE = 0.13, 95% CI (−0.01, 0.52), *p* = 0.053], with a moderate effect size (*d* = 0.42). Estimated marginal means suggested slightly higher post-test scores for the training group (*M* = 2.45) compared to the control group (*M* = 2.20).

**Table 5:** ANCOVA results with class random intercept.

Outcome	Predictor	Estimate	SE	95% CI	df	*t*	*p*
GEQ-MOD total (post)	Training	0.26	0.13	−0.00; 0.52	125	1.95	0.053
GRCS total (post)	Training	0.40	0.15	0.10; 0.71	125	2.60	0.010

Estimates derived from linear mixed models including training as fixed effect, baseline scores as covariates, and class as random intercept.

For gambling-related cognitions, the cluster-adjusted ANCOVA revealed a significant effect of training [*F*(1,125) = 6.77, *p* = 0.010]. Parameter estimates indicated that students in the intervention group reported higher post-test scores compared to those in the control group [*b* = 0.40, SE = 0.15, 95% CI (0.10, 0.71)], corresponding to a moderate effect size (*d* = 0.47). Students in the training group reported higher post-test scores (*M* = 2.25) than those in the control group (*M* = 1.85), when controlling baseline levels. Intraclass correlation coefficients were negligible for both outcomes (ICC < 0.001 for GEQ-MOD total and GRCS total), indicating minimal clustering at the classroom level.

Thematic analysis of the three focus groups identified three overarching themes that illustrate how students perceived and experienced the *Game in Lab* training (Table 6). To better align with the program objectives, themes were grouped as follows: Critical thinking (reflection on luck vs. strategy, understanding probability, repetition and attraction, comparison with traditional games); Emotional regulation (adrenaline and activation, anger and frustration, euphoria and involvment, and collaboration); Awareness of risk (speed of loss, Recognition of personal limits, illusion of control, change in perspective).

**Table 6:** Themes and subthemes emerging from the student focus groups with illustrative quotes.

Theme	Subtheme	Example quotes
Critical thinking	Reflection on luck vs. strategy	*“…being good at something does not really matter if you want to win.” / “I realized that you cannot do anything to change the result, you can only hope for luck.”*
	Understanding probability	*“At first some thought that if a 6 had just come out, it was less likely to come out again, but actually every roll has the same probability.” / “When you understand that the chances are the same, you realize that betting more does not help.”*
	Repetition and attraction	*“After losing I wanted to try again right away, even if I knew it was the same thing.” / “It seems like you have more chances if you play again, but in reality nothing changes.”*
	Comparison with traditional games	*“There is not the same strategy as in a normal board game, it’s more of a challenge with luck.”*
Emotional regulation	Adrenaline and activation	*“I felt the adrenaline rising, I do not know if it was positive or negative.” / “Every time I rolled the dice my heart was pounding.”*
	Anger and frustration	*“I have to win… I get really angry with these games.” / “When you lose after feeling sure, you get angry, you feel tricked.”*
	Euphoria and involvement	*“It was such incredible euphoria in that moment, and that’s when you lose, you are not rational.” / “When we won together, we felt super strong, as if we could not lose anymore.”*
	Solidarity and collaboration	*“The feeling of victory united us… we were very happy, very excited.” / “We encouraged each other, as if we were a team.”*
Awareness of risk	Speed of loss	*“It only took one bad throw to lose everything I had accumulated.”*
	Recognition of personal limits	*“I realized that I get excited too easily and I could be at risk.”*
	Illusion of control and repetition	*“If the first game went well, I would have bet more on the second, losing more.” / “After a win you convince yourself that luck will continue, but it does not.”*
	Change in perspective	*“I thought only older people would fall into it, but I found out it’s much easier to become addicted.” / “I understood that it’s not a matter of age but of how you let yourself get caught up in the game.”*

The first theme, critical thinking, captured students’ reflections on chance and probability. Participants reported becoming more aware that gambling outcomes are largely unpredictable and not influenced by personal strategies or rituals.

The second theme, emotional regulation, described students’ recognition of the emotional states elicited by gameplay (e.g., excitement, frustration, disappointment) and their perceived ability to manage these emotions more effectively after the training. Students described both the intensity of these emotions (e.g., “Every time I rolled the dice my heart was pounding”) and their consequences, such as feeling irrational when overly excited or angry when losing. At the same time, some participants emphasized experiences of solidarity and collaboration, highlighting how playing as a group fostered mutual encouragement and team spirit.

The third theme, awareness of risk, reflected an increased understanding of gambling-related risks. Students emphasized how the training helped them realize the ease of losing money or control when gambling, fostering more cautious attitudes toward gambling activities. Several participants noted a change in perspective, recognizing that gambling risks are not confined to older people but are relevant to their own age group.

Overall, these qualitative findings suggest that the Game in Lab training stimulated processes of cognitive and emotional reflection, which may represent important preventive mechanisms.

## Discussion

The results of this pilot study provide a detailed understanding of the preventive potential of board games in addressing adolescent gambling-related cognitions and expectancies. Quantitative analyses have shown mixed findings; while descriptive trends suggested reductions in gambling-related cognitions and expectancies across time, cluster-adjusted models did not confirm significant group differences, with intervention participants maintaining slightly higher post-test scores. Conversely, qualitative findings indicated meaningful cognitive and emotional reflections triggered by the training, pointing to a process of increased awareness rather than immediate behavioral change. These quantitative findings require different interpretation. Although descriptive trends suggested reductions over time, cluster-adjusted analyses did not confirm a statistically significant intervention effect on gambling expectancies and indicated higher adjusted post-test gambling-related cognitions in the training group compared to controls. These findings warrant careful interpretation and suggest that methodological and contextual factors should also be considered. One possible explanation is a short-term measurement reactivity effect (French and Sutton, 2010; Long et al., 2025), whereby participation in the intervention may have increased students’ awareness of gambling-related mechanisms, leading them to recognize and report such cognitions more readily at post-test. However, this interpretation remains speculative and should not be considered a definitive account of the observed pattern. Alternative explanations must also be considered. Although baseline scores were statistically controlled, initial group differences may have influenced post-test estimates, particularly in the context of a modest sample size and cluster-based design. Intraclass correlation coefficients derived from the mixed models were negligible (ICC < 0.001), indicating minimal clustering at the classroom level. While this suggests that classroom-level variance did not substantially influence the estimates, the limited number of clusters may still have reduced the statistical power to detect small intervention effects. In addition, participation in an interactive and reflective intervention may have heightened sensitivity to questionnaire content, potentially generating demand characteristics or increased salience of gambling-related constructs. Finally, the dosage of the intervention, three sessions delivered over a short period, may have been insufficient to produce measurable reductions in declarative cognitions as assessed by self-report scales. Taken together, these considerations suggest that the quantitative findings should be interpreted as preliminary and exploratory rather than conclusive evidence regarding intervention effectiveness.

Second, the timing of assessment might have influenced results. Data collection immediately followed the intervention, while processes such as critical reflection, emotional regulation, and risk awareness may require time and real-life experience to consolidate. In adolescent gambling prevention, follow-up assessments have captured changes that were not fully observable at immediate post-test, for example, a study reported reductions in gambling frequency and problem severity at 3–4-month follow-up after short-term cognitive gains (Donati et al., 2018). Similarly, research has shown that engagement in simulated gambling activities can predict later gambling with real money, underscoring the importance of early preventive reflection on such experiences (Hayer et al., 2018). Recent evidence further highlights how simulated play contexts may shape adolescents’ cognitive and emotional responses to risk, influencing both awareness and susceptibility to gambling-related mechanisms (Monson et al., 2024). More broadly, evidence from universal school-based programs indicates that cognitive and attitudinal effects can emerge or stabilize several months of post-intervention (Griffin et al., 2023). Therefore, future research should include longitudinal follow-ups to determine whether increased awareness translates into sustained cognitive change. Third, the limitations of self-report instruments must be acknowledged. Measures such as the GRCS and GEQ-MOD primarily capture declarative cognitions, which may not fully represent the complexity of reflective processes elicited by experiential learning. Integrating behavioral indicators (e.g., probabilistic reasoning tasks, simulated choices) or ecological momentary assessments could provide a more comprehensive understanding of cognitive and emotional shifts produced by the intervention.

Despite these limitations, qualitative findings offer valuable insights into how board game–based interventions can foster prevention processes during adolescence. Students described enhanced critical thinking about randomness and probability, emotional regulation during excitement and frustration, and increased awareness of risk related to control illusions and loss dynamics. These narratives suggest that the intervention activated mechanisms consistent with experiential learning theory and participatory health education models (Jones, 2017), emphasizing reflection-on-action as a key pathway for cognitive and emotional change. From this perspective, board games provide a safe and engaging context for adolescents to experience gambling-like emotions while deconstructing misconceptions through guided discussion.

The mixed-methods design of this study further strengthens these interpretations. Quantitative findings captured broad patterns of cognitive change, while qualitative data highlight the underlying reflective processes and meanings attributed by students. This triangulation underlines the value of combining self-report and narrative evidence to evaluate preventive interventions targeting complex cognitive constructs such as gambling distortions. Several methodological limitations should be acknowledged. First, the study relied on a relatively modest sample size and experienced substantial attrition, which may have reduced statistical power and limited the precision of effect estimates, despite additional analyses suggesting no systematic dropout bias. Second, outcomes were assessed exclusively through self-report measures. Such instruments may be influenced by social desirability, reporting bias, or heightened sensitivity to questionnaire content following participation in an educational intervention. Self-report scales may capture declarative beliefs rather than deeper cognitive restructuring or behavioral change. Third, assessment occurred immediately after the intervention, preventing evaluation of delayed or sustained effects. Cognitive and emotional processes activated through experiential learning may require time to consolidate and translate into measurable changes in gambling-related cognitions. The absence of follow-up assessments limits conclusions regarding the durability of potential effects. Fourth, although classes were randomized, contextual differences across school types (e.g., high schools, technical institutes, vocational schools) may have influenced engagement, classroom climate, and responsiveness to the intervention. These contextual factors were not explicitly modeled in the analyses and may have contributed to variability in outcomes. Finally, intervention sessions were delivered by trained facilitators, and potential facilitator-related effects (e.g., delivery style, relational dynamics, group management) were not formally assessed. Such variability may have influenced students’ engagement and responses, representing an additional source of unmeasured heterogeneity.

Future research should replicate these findings in larger samples, adopt multi-wave designs including follow-ups, and test behavioral or implicit measures of gambling cognition. Moreover, incorporating teachers as co-facilitators could enhance the ecological validity and sustainability of such interventions. Beyond gambling prevention, the training model proposed here, combining play, reflection, and collaborative learning, may also be adapted to other school-based interventions to promote healthier behaviors.

In conclusion, this pilot study suggests that board games can serve as a feasible and engaging tool to promote reflection on risk, probability, and self-regulation among adolescents. Although quantitative outcomes did not show immediate reductions in gambling cognitions, qualitative evidence indicates that experiential and participatory methods can activate critical awareness, an essential first step toward preventive change.

## Conclusion

This pilot study highlights the feasibility of using board games as a school-based tool for gambling prevention. Although quantitative outcomes did not demonstrate statistically robust reductions in gambling cognitions, qualitative results suggest that the training stimulated critical thinking, emotional awareness, and recognition of gambling risks. Future research with larger samples and follow-up assessments is needed to clarify the preventive potential of this approach.

## Data Availability

The raw data supporting the conclusions of this article will be made available by the authors, without undue reservation.
